# Association between place of birth and timely breastfeeding initiation among Cambodian women: a population-based study

**DOI:** 10.1186/s13006-022-00496-3

**Published:** 2022-07-23

**Authors:** Raleigh M. Harriott, Zelalem T. Haile, Ilana R. Azulay Chertok, Mohammad Rifat Haider

**Affiliations:** 1 College of Health Sciences and Professions, Ohio University, Athens, OH USA; 2 Department of Social Medicine, Heritage College of Osteopathic Medicine, Ohio University, Dublin, OH USA; 3 School of Nursing, College of Health Sciences and Professions, Ohio University, Athens, OH USA; 4 Department of Health Policy and Management, College of Public Health, University of Georgia, Athens, GA USA

**Keywords:** Breastfeeding initiation, Demographic and health survey, Cambodia

## Abstract

**Background:**

Enhancing timely breastfeeding initiation within the first hour postpartum is a goal the WHO’s Early Essential Newborn Care (EENC) and Baby-friendly Hospital Initiative (BFHI) aim to achieve globally. However, many health professionals and facilities have yet to adopt these guidelines in Cambodia, impeding timely initiation progress and maternal-infant health goals.

**Methods:**

This secondary data analysis used the 2014 Cambodia Demographic and Health Survey (CDHS) data of 2,729 women who gave birth in the two years preceding the survey to examine the association between place of birth and timely breastfeeding initiation. Descriptive statistics, chi-square test and multivariable logistic regression were performed. Pairwise interaction terms between place of birth and each covariate were included in the regression model to examine the presence of multiplicative effect modification.

**Results:**

The prevalence of timely breastfeeding initiation was 62.9 percent. Most women gave birth in public health facilities (72.8%) followed by private health facilities (15.9%) and at home (11.2%). The proportions of timely breastfeeding initiation differ by place of birth (*p* < 0.001). In the multivariable model, there was a significant interaction between place of birth and household wealth index and between place of birth and residence on timely initiation. Among women who reside in poor households, the odds of timely initiation were lower among women who gave birth at home compared to those who gave birth in public health facilities, adjusted odds ratio (95% confidence interval) 0.43 (0.21, 0.88). For urban settings, the odds of timely breastfeeding initiation were lower among women who gave birth in private health facilities compared to those who gave birth in public health facilities 0.52 (0.36, 0.75). For rural settings, the odds of timely breastfeeding initiation were lower among women who gave birth at home compared to those who gave birth in public health facilities 0.55 (0.31, 0.97).

**Conclusions:**

Wealth index and residence moderated the association between place of birth and timely breastfeeding initiation in Cambodia. To improve breastfeeding outcomes and eliminate practices impeding timely initiation, breastfeeding advocacy programs need greater integration and follow-up in Cambodia’s health systems, including among home birth attendants and private health facilities.

**Supplementary Information:**

The online version contains supplementary material available at 10.1186/s13006-022-00496-3.

## Background

The risk of not breastfeeding contributes to detrimental health and economic outcomes for infants, mothers, and communities [1–3]. Initiating breastfeeding in the first few moments of life, builds the foundation for adequate and continued breastfeeding practices. Breastmilk contains vital nutrients for optimal infant health and child growth. The health, development, and survival of infants, children, and mothers are significantly impaired when adequate breastfeeding is not practiced [2]. The World Health Organization (WHO) and United Nations Children’s Fund (UNICEF) recommend initiating breastfeeding within the first hour after birth (“timely initiation”), exclusive breastfeeding for the first six months of life, and continued breastfeeding for up to two years or beyond, with the addition of safe and adequate complimentary foods [2, 4], yet globally, only 44% of infants are breastfed within the first hour [2].

Timely breastfeeding initiation ensures the provision of early milk, colostrum, which offers vital nutrients specific to each infant [5]. Moreover, colostrum is rich in immunoglobins, providing greater resistance to diseases, including viral, bacterial, fungal, and protozoa infections [5]. Timely breastfeeding initiation and immediate skin-to-skin contact (SSC) support infant cardio-respiratory system during the transition to extra-uterine life [6]. In a dose–response effect, SSC after birth contributes to improved breastfeeding outcomes [7].

Delayed breastfeeding can have negative consequences, significantly increasing the risk of neonatal mortality [8], whereas timely initiation is associated with a 44% lower risk of mortality and a 42% reduction in mortality risk among low-birthweight infants [9]. A delay in breastfeeding of 2 to 23 h postpartum was associated with an increased risk of neonatal mortality by 33% compared to infants initiating within one hour [10].

Research regarding timely breastfeeding initiation and associated factors of Cambodian women is lacking. Globally, associated factors influencing timely initiation include smoking, parity, mode of birth, dyad separation, and maternal education [11]. Positive dyad connections, including early SSC and rooming-in, are associated with an increase in timely breastfeeding initiation and breastfeeding continuation [11]. Timely breastfeeding initiation stimulates lactation via infant suckling and oxytocin secretion [12] and can be offered on-demand by rooming infant and mother together. Effectively training health professionals to practice and promote the WHO/UNICEF guideline of breastfeeding within the first hour of life will facilitate the goal in diverse birthing settings.

East Asian countries and the Pacific report the lowest timely breastfeeding initiation rates (32%) globally [7, 13]. To address this disparity, the WHO launched the Action Plan for Healthy Newborn Infants in the Western Pacific Region (2014–2020), strategically focusing on quality improvement of early essential newborn care (EENC) and access to quality skilled birth and newborn infant care [14]. EENC improves birth practices including timely breastfeeding initiation, eliminating unnecessary early dyad separation [7], offering ample SSC time of at least 90 min [6], thereby supporting early breastfeeding when infants exhibit first-feeding cues [15]. Early essential newborn care implementation began in eight countries: Cambodia, China, Lao People’s Democratic Republic, Mongolia, Papua New Guinea, Philippines, Solomon Islands and Vietnam, which collectively account for over 95% of neonatal deaths in east Asia and the Pacific [7]. EENC outlines a national and subnational health agenda, including a health professionals’ support plan to ensure its adoption and implementation at every birth [14].

Globally, sociodemographic factors associated with not initiating breastfeeding within one hour include low family income, low maternal education, and home birth [16]. Higher maternal education compared to the lowest level, was associated with higher likelihood of timely breastfeeding initiation, while lower maternal age and household income were associated with a decreased timely initiation [11]. Only 30.3% of mothers reported receiving messages about timely breastfeeding initiation during their antenatal care visits and 32.3% received exclusive breastfeeding messages, while 18.4% of women reported receiving recommendations to use breastmilk substitutes from health professionals [17].

Marketing of breast-milk substitutes remains rampant in east Asia and the Pacific, impeding breastfeeding progress [18] and timely initiation. Breastmilk substitutes, like prelacteal feeds, inhibit the nutritional benefits of colostrum and potentially weaken the maternal-infant bond in the first moments and days of breastfeeding. Breastmilk substitutes have traditionally been sweetened condensed milk or other canned milk, thinned with water, watery rice porridge, and/or infant formula [19]. Offering any food or drink other than breastmilk in the first days of life can increase infant morbidity and reduce the duration and exclusivity of breastfeeding [20]. Use of prelacteal feeds in Cambodia dramatically decreased from 2000 (94.5%) to 2010 (19.1%), likely due to extensive public health efforts and breastfeeding campaigns [21], however the rate increased in 2014 to 27.7% [19]. It should be noted that prelacteal feed increases from 2010 to 2014, coincide with a rise in facility-based births in Cambodia, 54% in 2010 [22] to 82% in 2014 [23]. A 2016 study conducted in Phnom Penh, the capital and most populous city in Cambodia, revealed 43.1% of children age 0 to 5 months consumed breastmilk substitutes [17]. Breastmilk substitutes remain pervasive among Cambodian infant feeding practices.

From 2000 to 2010, rates of timely breastfeeding initiation increased in Cambodia from 11 to 66%, respectively [24]. However, the 2014 CDHS recently reported a slight drop in timely breastfeeding initiation rates at 63 percent [19]. The recent decline in timely initiation rates [24] and lack of support by healthcare providers [17] indicate a need for increased and sustained breastfeeding advocacy and buy-in among health professionals and facilities. In addition, the recent overhaul of Cambodia’s healthcare system aims to address inequalities among competing private and public sectors. Cambodia’s private sector largely favors the wealthiest quintile [25] and is typically the first point of contact with 65% of rural residents and 67–78% of urban residents choosing private providers [26, 27]. Furthermore, place of birth can impact breastfeeding practices as health professional attitudes can vary by facility type: public, private, or home visits.

The WHO’s Baby-friendly Hospital Initiatives (BFHI) have been generally supported, however, many health professionals object to specific steps, such as immediate SSC, feeling that exposing an infant is contraindicated based on their training and experience [28]. Rooming-in has also been challenged, citing lack of space for the infant crib [28]. Lack of buy-in for these and other BFHI steps is more likely to be reported by older generation health professionals [28]. In addition, one of the major barriers to implementation of BFHI, is the incentivized relationship between breast-milk substitute companies, decision makers, and health professionals [28]. The 2017 *National Implementation Report* cites many countries have terminated the BFHI program due to lack of external and/or government funding, political interest, human resources, resistance from hospitals or healthcare systems, adoption of other initiatives, and non-adherence to the International Code of Marketing of Breast-milk Substitutes [28].

Timely breastfeeding initiation in Cambodia remains low. While breastmilk substitute promotion persists [17] and BFHI compliance assessment is lacking [28, 29], health professionals and facilities can protect breastfeeding by promoting SSC, rooming-in, recruiting government support, and offering lactation counseling [30, 31]. With two-thirds of public health professionals working privately in Cambodia [26], crossover training has greater potential across sectors for breastfeeding advocacy. Therefore, place of birth and the healthcare team can influence timely initiation. This study was conducted to examine the association of place of birth on timely breastfeeding initiation among Cambodian women.

## Methods

### Data

Secondary data analysis was conducted using the 2014 CDHS data. The 2014 CDHS is the fourth consecutive cross-sectional survey administered in Cambodia with support from multiple local and international organizations [19]. The CDHS interviews women and men between the ages of 15 and 49 and stands as a national representative sample of 19 designated sampling domains, including separate indicators for rural and urban areas. The CDHS sampling frame was derived from the list of enumeration areas (EAs) that were defined for the 2008 Cambodia General Population Census that was updated in 2012 and implemented by the National Institute of Statistics. The survey used a two-stage stratified sample, by separating domains into 24,210 rural or 4,245 urban clusters, respectively. In the first stage, the probability proportional to size technique was used to select a total of 611 EAs including 188 EAs in urban and 423 EAs in rural areas. In the second stage, an equal probability systematic sampling technique was used to select 24 households from every urban EAs and 28 households from every rural EAs. Women aged 15–49 who were either resident of the selected households or visitors present in the household on the night before the survey were eligible to be interviewed [14].

**Fig. 1:**
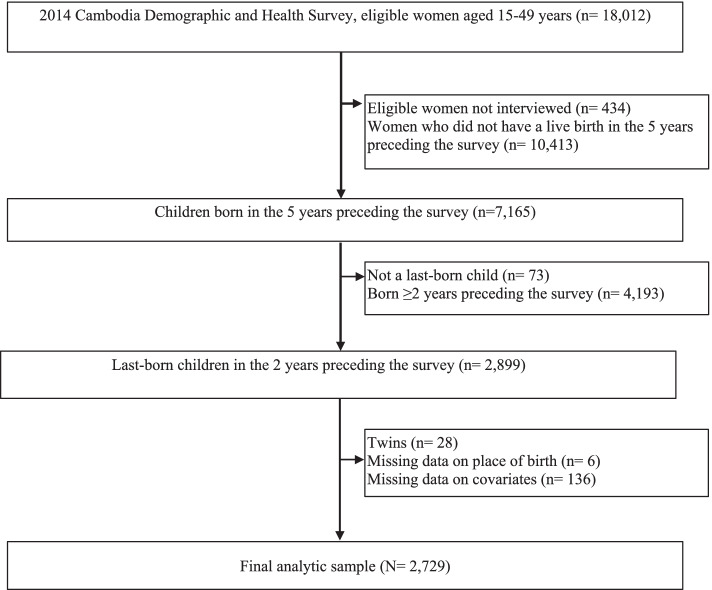
Study sample selection

In the 2014 CDHS, a total of 7,165 women had a live birth in the five years preceding the survey. The current study was restricted to the last-born children in the two years preceding the survey (*n* = 2,899). We further excluded twins (*n* = 28) and participants with missing data on place of birth (*n* = 6) and other covariates assessed in the current study (*n* = 136). The final sample for this study consisted of 2,729 children (Fig. 1).

The 2014 CDHS was granted permission by the government of Cambodia, maintained participant confidentiality, and used informed consent before conducting the survey [19]. This study was approved by the DHS, allowing data to be downloaded and used for analysis. This study was exempt from full institutional review board review, as it analyzed an existing dataset with unidentifiable participant information.

The CDHS questionnaires were translated from English to Khmer, Cambodia’s national language. Questionnaires included questions regarding participants’ background and sociodemographic characteristics; for the full list see the CDHS reference [19]. The CDHS questionnaire was pretested and transmuted to reflect relevant health issues specific to Cambodia’s population.

### Measures

The dependent variable was timely breastfeeding initiation defined based on the question, “How long after birth did you first put the index child to the breast?” Responses include immediately, within the first hour, more than the first hour, and days after birth. For analysis, this variable was dummy coded so that women who responded “immediately” and “within the first hour” were coded as “yes”, and mothers who responded more than one hour after birth were coded as “no.” Respondents with missing data on timely initiation (*n* = 2) were excluded from the analysis and women who never breastfed were also excluded from the analysis (*n* = 119).

The independent variable, place of birth was defined as home (respondent’s home or other homes), public sector (government hospital, government health center, government health post or other public sectors), or private medical sector (private hospital or clinic, other private medical sectors).

The following covariates were adjusted in the multivariable model: maternal age (15–24, 25–35, 36–49), maternal education (none, primary, secondary, or above), marital status (married/living together, or divorced/widowed/separated, “not married”), household wealth index (lowest and second were combined as “poor,” middle remained “middle,” fourth and highest were combined as “rich”), parity (primiparous for first birth or multiparous for two or more births), child’s sex (male or female), type of place of residence (urban or rural), maternal employment status (no or yes), health insurance (no or yes), number of antenatal care visits during pregnancy (0, 1–3, 4 +), cesarean birth (no or yes), type of birth attendant (doctor, nurse/midwife, or traditional birth attendant/other), and prelacteal feed, defined by the CDHS as something other than breastmilk given to the infant in the first three days of life (no or yes).

### Statistical analysis

Frequencies and proportions were used to describe the characteristics of the study sample. Rao-Scott chi-square test statistic (χ2) was used to compare timely breastfeeding initiation by the place of birth and each covariate. Unadjusted logistic regression analyses were performed to determine the independent association between place of birth and timely breastfeeding initiation. All variables were retained in the multivariable model regardless of statistical significance. Pairwise interactions between place of birth and each covariate were performed to examine differences in the relationship between place of birth and timely breastfeeding initiation across different categories of covariates. There were significant interactions between place of birth and household wealth index and between place of birth and residence on timely breastfeeding initiation. Multivariable adjusted logistic regression models were performed to examine the independent association of place of birth on timely breastfeeding initiation, stratified by household wealth index and residence. Odds ratio (OR) and 95% confidence intervals (CI) were determined. Complex sample design elements including stratification, cluster and weights were incorporated in all analyses. *P* < 0.05 was considered statistically significant. All analyses were performed using SAS OnDemand for Academics (SAS Institute, Cary, NC).

## Results

Among the total sample of 2,729 women, 96.4% were married or living with their partner, 51.4% were aged 24–34, 50.6% had at least a primary level of education, and 86.5% resided in rural provinces of Cambodia. The majority of women had a nurse or midwife assist with their birth (75.6%), gave birth in public health facilities (72.8%), and reported four or more antenatal care visits throughout pregnancy (76.0%). The prevalence of timely breastfeeding initiation was 65.2% and 27.3% of women reported offering prelacteal feeds (Table 1).

**Table 1:** Descriptive statistics of the study sample (*N* = 2729)

	***n* (Wt. %)**
**Mother’s age**	
15–24	1020 (37.9)
24–34	1424 (51.4)
35–49	285 (10.7)
**Mother’s education**	
None	359 (12.2)
Primary	1348 (50.6)
Secondary or above	1138 (37.1)
**Marital status**	
Married/living with partner	2627 (96.4)
Not married	102 (3.6)
**Household wealth index**	
Poor	1201 (43.4)
Middle	441 (19.2)
Rich	1203 (37.4)
**Parity**	
Primiparous	1123 (41.0)
Multiparous	1606 (58.9)
**Child’s sex**	
Male	1372 (50.4)
Female	1357 (49.6)
**Residence**	
Urban	725 (13.5)
Rural	2004 (86.5)
**Employment**	
No	1141 (44.5)
Yes	1588 (55.5)
**Health insurance**	
No	2309 (83.1)
Yes	420 (16.9)
**Antenatal visits during pregnancy**	
None	129 (3.6)
1–3 visits	567 (20.4)
4 or more visits	2033 (76.0)
**Cesarean birth**	
No	2521 (92.7)
Yes	208 (7.3)
**Type of birth attendant**	
Doctor	425 (17.2)
Nurse/midwife	2045 (75.6)
TBA or other	259 (7.2)
**Prelacteal feeds**	
No	2068 (72.7)
Yes	661 (27.3)
**Timely breastfeeding initiation**	
No	1012 (34.8)
Yes	1717 (65.2)
**Place of birth**	
Public facility	1985 (72.8)
Private facility	396 (15.9)
Home	348 (11.2)

*Abbreviations: Wt.%* Weighted percent, *TBA* Traditional birth attendant

In bivariate analyses, the timely breastfeeding initiation rates differed by place of birth. Rates were higher among women who gave birth in public facilities than private facilities or at home (70.1% vs. 53.5% vs. 50.5%; *p* < 0.001) (Table 2). Other factors significantly associated with timely initiation included education level, marital status, household wealth index, parity, residence, employment, and mode of birth.

**Table 2:** Characteristics of the study sample by timely breastfeeding initiation (N = 2729)

	**Timely Breastfeeding Initiation**				
	**No *n*(Wt. %)**	**Yes *n*(Wt.%)**	***P***	**Unadjusted OR (95% CI)**	***P***
**Mother's age**			0.326		
15–24	370 (33.4)	650 (66.6)		Reference	
24–34	535 (36.5)	889 (63.5)		0.87 (0.69, 1.10)	0.258
35–49	107 (31.6)	178 (68.4)		1.09 (0.77, 1.55)	0.641
**Mother's education**			0.004		
None	159 (39.5)	188 (60.5)		Reference	
Primary	430 (30.8)	866 (69.2)		1.47 (1.06, 2.03)	0.021
Secondary or above	423 (38.7)	663 (61.3)		1.03 (0.75, 1.43)	0.837
**Marital status**			0.031		
Married/living with partner	965 (34.3)	1662 (65.7)		Reference	
Not married	47 (47.9)	55 (52.0)		0.57 (0.33, 0.96)	0.034
**Household wealth index**			0.006		
Poor	440 (35.2)	726 (64.8)		Reference	
Middle	137 (27.4)	294 (72.6)		1.44 (1.07, 1.94)	0.016
Rich	435 (38.2)	697 (61.8)		0.88 (0.69, 1.12)	0.297
**Parity**			0.005		
Primiparous	451 (38.7)	672 (61.3)		Reference	
Multiparous	561 (32.0)	1045 (67.9)		1.34 (1.09, 1.65)	0.006
**Child’s sex**			0.108		
Male	524 (36.6)	848 (63.4)		Reference	
Female	488 (32.9)	869 (67.1)		1.18 (0.96, 1.45)	0.109
**Residence**			< .001		
Urban	278 (44.8)	447 (55.2)		Reference	
Rural	734 (33.2)	1270 (66.7)		1.63 (1.27, 2.09)	0.0001
**Employment**			0.004		
No	374 (31.1)	767 (68.8)		Reference	
Yes	638 (37.7)	950 (62.3)		0.75 (0.61, 0.91)	0.004
**Health insurance**			0.305		
No	869 (35.4)	1440 (64.6)		Reference	
Yes	143 (31.6)	277 (68.4)		1.19 (0.85, 1.65)	0.309
**Antenatal visits during pregnancy**			0.327		
None	71 (42.7)	58 (57.3)		Reference	
1 to 3	214 (36.3)	353 (63.7)		1.31 (0.76, 2.26)	0.337
4 or more	727 (33.9)	1306 (66.0)		1.45 (0.88, 2.39)	0.148
**Cesarean birth**			< .001		
No	866 (31.9)	1655 (68.1)		Reference	
Yes	146 (71.2)	62 (28.8)		0.19 (0.13, 0.29)	< .001
**Type of birth attendant**			< .001		
Doctor	244 (50.2)	216 (49.8)		Reference	
Nurse/Midwife	731 (32.3)	1389 (67.7)		2.22 (1.76, 2.79)	< .001
TBA/other	153 (55.2)	112 (44.8)		0.86 (0.54, 1.37)	0.528
**Prelacteal feeds**			< .001		
No	656 (28.4)	1412 (71.6)		Reference	
Yes	356 (51.8)	305 (48.2)		0.37 (0.29, 0.47)	< .001
**Place of birth**			< .001		
Public	643 (29.9)	1342 (70.1)		Reference	
Private	188 (46.5)	208 (53.5)		0.49 (0.38, 0.64)	< .001
Home	181 (49.5)	167 (50.5)		0.44 (0.31, 0.61)	< .001

*P*-values are derived from Rao-Scott Chi-square test

In the multivariable model, there were significant interactions between place of birth and household wealth index (Interaction *p* = 0.021) and between place of birth and residence (Interaction *p* = 0.021) on timely breastfeeding initiation. Table 3 displays the association between place of birth and timely initiation stratified by household wealth index. Among women residing in poor household wealth index, the odds of timely initiation were 57% lower for those who gave birth at home compared to those who gave birth at public facilities (0.43; 0.21, 0.88; *p* = 0.020). For women residing in the middle and rich household wealth index, no associations were detected between place of birth and timely breastfeeding initiation.

**Table 3:** Association between sociodemographic variables with timely breastfeeding initiation stratified by household wealth index (N = 2729)

	**Poor (*n* = 1166)**		**Middle (*n* = 431)**		**Rich (*n* = 1132)**	
	**AOR (95% CI)**	***P***	**AOR (95% CI)**	***P***	**AOR (95% CI)**	***P***
**Mother's age**						
15–24	Reference		Reference		Reference	
24–34	0.86 (0.58, 1.27)	0.439	0.82 (0.44, 1.52)	0.519	0.71 (0.44, 1.13)	0.147
35–49	0.95 (0.56, 1.63)	0.853	1.85 (0.56, 6.09)	0.311	1.43 (0.62, 3.31)	0.402
**Mother's education**						
None	Reference		Reference		Reference	
Primary	1.26 (0.83, 1.92)	0.285	0.86 (0.33,2.21)	0.749	2.59 (0.77,8.79)	0.125
Secondary or above	1.39 (0.83, 2.33)	0.209	0.81 (0.29, 2.23)	0.686	1.69 (0.51, 5.64)	0.391
**Marital status**						
Married/living together	Reference		Reference		Reference	
Not married	0.53 (0.26,1.06)	0.073	0.88 (0.23, 3.36)	0.854	0.46 (0.22, 0.96)	0.146
**Parity**						
Primiparous	Reference		Reference		Reference	
Multiparous	1.44 (0.97, 2.15)	0.073	1.42 (0.73, 2.76)	0.296	1.25 (0.81, 1.94)	0.314
**Child’s sex**						
Male	Reference		Reference		Reference	
Female	1.29 (0.93, 1.79)	0.126	1.48 (0.92, 2.40)	0.107	1.09 (0.78, 1.51)	0.625
**Residence**						
Urban	Reference		Reference		Reference	
Rural	1.10 (0.45, 2.67)	0.833	0.63 (0.24, 1.66)	0.349	1.26 (0.91, 1.73)	0.161
**Employment**						
No	Reference		Reference		Reference	
Yes	0.73 (0.53, 1.00)	0.052	0.83 (0.48, 1.45)	0.515	0.79 (0.57, 1.11)	0.169
**Health insurance**						
No	Reference		Reference		Reference	
Yes	1.02 (0.63, 1.64)	0.944	0.99 (0.42, 2.33)	0.99	1.26 (0.71, 2.25)	0.429
**Antenatal visits during pregnancy**						
None	Reference		Reference		Reference	
1 to 3	0.96 (0.52, 1.79)	0.896	0.44 (0.07, 2.92)	0.397	1.96 (0.45, 8.57)	0.370
4 or more	0.98 (0.53, 1.81)	0.952	0.47 (0.08, 2.63)	0.385	1.69 (0.44, 6.54)	0.446
**Cesarean birth**						
No	Reference		Reference		Reference	
Yes	0.35 (0.13, 0.95)	0.039	0.13 (0.03, 0.50)	0.003	0.26 (0.15, 0.47)	< .001
**Type of birth attendant**						
Doctor	Reference		Reference		Reference	
Nurse/Midwife	1.09 (0.49, 2.42)	0.822	0.90 (0.36, 2.26)	0.828	1.04 (0.68, 1.61)	0.850
TBA/other	0.87 (0.27, 2.74)	0.805	0.94 (0.07, 12.55)	0.962	1.07 (0.18, 6.27)	0.939
**Prelacteal feeds**						
No	Reference		Reference		Reference	
Yes	0.39 (0.25, 0.59)	< .001	0.48 (0.26, 0.88)	0.018	0.51 (0.35, 0.72)	< 0.001
**Place of birth**						
Public	Reference		Reference		Reference	
Private	0.79 (0.38, 1.60)	0.505	0.67 (0.29, 1.56)	0.351	0.86 (0.61, 1.20)	0.376
Home	0.43 (0.21, 0.88)	0.020	0.33 (0.05, 1.97)	0.219	1.59 (0.61, 4.15)	0.347

*Abbreviations: AOR* Adjusted Odds Ratio, *CI* Confidence Interval

In the stratified analyses by household wealth index (Table 3), other factors that were significantly associated with timely breastfeeding initiation included mode of birth, prelacteal feeds, and parity. Compared to vaginal birth, women who gave birth via cesarean were less likely to timely initiate breastfeeding across the different household indices: women from a poor household wealth index were 65% less likely (0.35; 0.13, 0.95; *p* = 0.039), women from a middle household wealth index were 87% less likely (0.13; 0.03, 0.50; *p* = 0.003), and those from a rich household wealth index were 74% less likely (0.26; 0.15, 0.47; *p* =  < 0.001).

Women who offered prelacteal feeds had lower odds of timely breastfeeding initiation across all household wealth indices compared to women who did not offer prelacteal feeds: poor (0.39; 0.25, 0.59; *p* < 0.001), middle (0.48; 0.26, 0.88; *p* = 0.018), rich (0.51; 0.35, 0.72; *p* < 0.001). Multiparous women had greater odds of timely breastfeeding initiation compared to primiparous women of poor household wealth index (1.44; 0.97, 2.15; *p* = 0.073), while no significant association was found among women residing in middle and rich household wealth indices.

Table 4 presents the results of analyses examining the association between place of birth and timely breastfeeding initiation, stratified by place of residence. Among women residing in urban settings, the odds of timely initiation were 48% lower for women who gave birth at a private health facility compared to a public facility (0.52; 0.36, 0.75; *p* = 0.001). Additional variables associated with lower odds of timely breastfeeding initiation for women of urban settings include having cesarean birth compared to vaginal birth (0.10; 0.05, 0.19; *p* < 0.001), practicing prelacteal feeds compared to not practicing and prelacteal feeds (0.28; 0.19, 0.42; *p* < 0.001), not married compared to married/living together (0.37; 0.14, 0.98; *p* = 0.045). On the other hand, additional variables associated with higher odds of timely breastfeeding initiation for women of urban settings include residing in middle household wealth index compared to poor household wealth index (3.64; 1.11, 11.94; *p* = 0.033), 1–3 antenatal care visits (7.58; 1.74, 32.9; *p* = 0.007) and 4 + visits (8.09; 2.27, 28.9; *p* = 0.001) compared to no antenatal care visits and having a traditional birth attendant compared to having a doctor as a birth attendant (9.03; 1.39, 58.8; *p* = 0.021).

**Table 4:** Association between sociodemographic variables with timely breastfeeding initiation stratified by residence (*N* = 2729)

	**Urban (*n* = 725)**		**Rural (*n* = 2004)**	
	**AOR (95% CI)**	***P***	**AOR (95% CI)**	***P***
**Mother's age**				
15–24	Reference		Reference	
24–34	0.69 (0.37, 1.29)	0.243	0.83 (0.62, 1.09)	0.191
35–49	1.29 (0.50, 3.34)	0.588	1.14 (0.72, 1.79)	0.586
**Mother's education**				
None	Reference		Reference	
Primary	0.87 (0.27, 2.86)	0.819	1.34 (0.94, 1.92)	0.108
Secondary or above	0.64 (0.18, 2.26)	0.484	1.18 (0.79, 1.75)	0.413
**Marital status**				
Married/living together	Reference		Reference	
Not married	0.37 (0.14, 0.98)	0.045	0.64 (0.36, 1.16)	0.144
**Parity**				
Primiparous	Reference		Reference	
Multiparous	1.05 (0.59, 1.84)	0.872	1.43 (1.07, 1.91)	0.015
**Child’s sex**				
Male	Reference		Reference	
Female	1.36 (0.85, 2.16)	0.200	1.24 (0.98, 1.57)	0.074
**Household wealth index**				
Poor	Reference		Reference	
Middle	3.64 (1.11, 11.94)	0.033	1.43 (1.04, 1.98)	0.029
Rich	1.92 (0.75, 4.89)	0.173	1.26 (0.89, 1.76)	0.186
**Employment**				
No	Reference		Reference	
Yes	0.97 (0.65, 1.43)	0.868	0.76 (0.59, 0.97)	0.024
**Health insurance**				
No	Reference		Reference	
Yes	1.10 (0.55, 2.20)	0.781	1.04 (0.71,1.51)	0.858
**Antenatal visits during pregnancy**				
None	Reference		Reference	
1 to 3	7.58 (1.74, 32.9)	0.007	0.83 (0.46, 1.49)	0.526
4 or more	8.09 (2.27, 28.9)	0.001	0.84 (0.48, 1.47)	0.529
**Cesarean birth**				
No	Reference		Reference	
Yes	0.10 (0.05, 0.19)	< .001	0.31 (0.18, 0.54)	< .001
**Type of birth attendant**				
Doctor	Reference		Reference	
Nurse/Midwife	0.97 (0.55, 0.19)	0.943	1.09 (0.75, 1.57)	0.657
TBA/other	9.03 (1.39, 58.8)	0.021	0.67 (0.29, 1.51)	0.336
**Prelacteal feeds**				
No	Reference		Reference	
Yes	0.28 (0.19, 0.42)	< .001	0.49 (0.37, 0.65)	< .001
**Place of birth**				
Public	Reference		Reference	
Private	0.52 (0.36, 0.75)	0.001	0.88 (0.61, 1.29)	0.517
Home	0.37 (0.09, 1.63)	0.188	0.55 (0.31, 0.97)	0.039

*Abbreviations: AOR* Adjusted Odds Ratio, *CI* Confidence Interval

Among women residing in rural settings, the odds of timely breastfeeding initiation were 45% lower among women who gave birth at home compared to public facilities (0.55; 0.31, 0.97; *p* = 0.039). For women of rural settings, additional variables associated with lower odds of timely breastfeeding initiation included having cesarean birth compared to vaginal birth (0.31; 0.18, 0.54; *p* < 0.001), and practicing prelacteal feeds compared to not practicing prelacteal feeds (0.49; 0.37, 0.65; *p* < 0.001) and being employed compared to unemployed (0.76; 0.59, 0.97; *p* = 0.024). On the other hand, additional variables associated with higher odds of timely breastfeeding initiation for women of rural settings include residing in middle household wealth index compared to poor household wealth index (1.43; 1.04, 1.98; *p* = 0.029) and being multiparous compared to primiparous (1.43;1.07, 1.91; *p* = 0.015).

## Discussion

While breastfeeding initiation within one hour of birth has increased in Cambodia since 2000 [21], additional efforts to facilitate timely initiation should be focused on women with noted barriers. Among women who reside in poor households, the likelihood of timely breastfeeding initiation was lower in women who gave birth at home compared to those who gave birth in public health facilities. Urban women giving birth in private facilities were less likely to timely initiate breastfeeding than urban women giving birth in public facilities, and women who gave birth in private facilities had greater rates of offering prelacteal feeds than women who gave birth in public facilities. These results suggest public health facilities and their health professionals are more likely to promote breastfeeding initiation than private facilities and home birth attendants.

A cross-sectional study found that 45.1% of mothers in Phnom Penh reported seeing breastmilk substitute advertisements in health facilities [20]. Violations of the Code are reported among private health facilities [28], as no monitoring system was implemented, and no penalties or fines were imposed on violators of the Code in Cambodia [29]. The 2015 Cambodia World Breastfeeding Trends Initiative (WBTi) reports the most popular private maternity facility distributes free infant formula [29]. Due to the weak monitoring of infant formula companies and their health facility partners, formula promotion violating the Code goes unrecognized and unreported [29]. The Cambodia WBTi designated only 47% of hospitals as Baby-friendly in the past five years, all of which were public health facilities [29], further depicting the influence place of birth can have on timely breastfeeding initiation.

As private facilities are concentrated in urban areas [32] and Phnom Penh is the most populous, urban city in Cambodia, women giving birth in urban private settings were possibly more exposed to breastmilk substitutes from infant formula companies and their health professional partners [28, 29], than women giving birth in rural private facilities. This trend may also contribute to the lack of BFHI buy-in in the private facilities [28] and to the discrepancy in timely breastfeeding initiation. Even though Cambodia’s commitment to universal healthcare has made recent progress, the private sector is skewed towards the richest quintile [25], while rural health centers tend to service the poor [32]. The Cambodian Ministry of Health has appropriated more medical staff, mainly nurses and midwives, to health centers in rural provinces and remote areas which had faced healthcare disparities due to low salaries and intense work conditions [32]. Facilitating partnerships among private and public sectors will grant more equal access to quality healthcare [25, 32, 33], as well as communicating current evidence-based breastfeeding care and programs.

These findings correspond with the increasing trend of breastmilk substitute use and its marketing, especially among women who give birth in private facilities. Previous findings show that 26.1% of women who gave birth in private facilities provided their infants younger than 6 months of age with breastmilk substitutes, five times more than women who gave birth in public facilities [21]. No association was found among rural women giving birth in private facilities on timely breastfeeding initiation, suggesting rural women may have a lower exposure to breastmilk substitutes compared to urban women in private facilities, thereby reducing their risk of impeding timely initiation by offering breastmilk substitutes. Rural women giving birth at home were less likely to timely initiate breastfeeding than urban women giving birth at home. This may be due to education or local community practices and beliefs [34], and family influences, as many women reported their mother or sisters were their main sources of breastfeeding advice at home [35]. In contrast, there was no significant association among urban women giving birth at home and timely initiation. As ‘urban’ indicates a modern, city setting, women residing in urban areas may forgo traditional postpartum practices.

Midwives and traditional birth attendants typically assist with home births [36], and in Cambodia, midwives are especially utilized in health centers [32]. Because of their role in antenatal care, midwives’ attitudes and actions can influence new mothers’ decisions to timely initiate breastfeeding. Midwives reported encouraging mothers to breastfeed after birth, however, timely breastfeeding was not observed in the first hour postpartum which may be explained by maternal feelings of modesty in the presence of an observer and a sense of discomfort discussing breastfeeding by younger midwives [35]. To encourage midwives and TBAs, Cambodia launched the Government Midwifery Incentive Scheme (GMIS) in 2015, providing cash incentives for midwives and other trained health personnel per live birth they attended in health facilities, with higher pay for health center births in rural areas [37]. GMIS helped decrease maternal mortality rates from 473 in 2005 to 206 in 2010 by increasing health facility births [19, 37]. Government Midwifery Incentive Scheme success in Cambodia, proposes performance-based incentive programs may also be an effective tool in improving related indicators of breastfeeding initiation, such as prolonged SSC following birth, assisting with timely breastfeeding initiation, and providing lactation support to new mothers. Reproductive, maternal, newborn and child health (RMNCH) programs [38], like EENC and GMIS, aim to improve healthcare financing and workforce in Cambodia, including universal healthcare coverage, increasing staff, and providing cash incentives [25, 32].

From 2000 to 2014, public health facility births increased in Cambodia by 82 percent [23]. The growing trend of exclusivity and timely breastfeeding initiation in Cambodia may be correlated with public health facilities that adopted the WHO/ UNICEF BFHI [22]. However, as previously mentioned, many countries have halted BFHI programs [28], or have merged or adopted the EENC Action Plan [14]. Cambodia is one of two countries achieving the Action Plan target of 80% of facilities introducing EENC, including immediate and sustained skin-to-skin contact [18]. Over half of hospitals reported forming EENC teams, however, only 19% reported conducting essential, routine quality care assessments, suggesting the need to gain health professionals’ and facilities’ support for the program.

The findings of the current study highlight the importance of educating and incentivizing health professionals to promote and regularly assess breastfeeding support, specifically those assisting private facility or home births. Support of timely initiation and lactation guidance extends beyond verbal encouragement [2, 35], therefore, promotion of EENC, for example, can improve timely initiation rates, breastfeeding exclusivity, and duration in Cambodia [18].

Women with cesarean births had lower odds of timely breastfeeding initiation regardless of household wealth index, with lower odds of timely breastfeeding initiation, compared to women who gave birth vaginally (65% among poor, 87% among middle, and 74% among rich). Efforts to support women post-cesarean should be implemented across all socioeconomic groups. Results are corroborated by previous research finding decreased odds of timely breastfeeding initiation among women who had cesarean births in low- and middle-income countries [39, 40]. Delayed initiation may be due to post-cesarean pain or fatigue, difficulty with breastfeeding positioning related to the incision site, and maternal-infant separation [39, 41, 42].

Multiparous women and those who reside in middle wealth index households were more likely to timely initiate breastfeeding. Multiparous initiation rates are consistent with other southeast Asia studies suggesting women have greater self-efficacy based on previous breastfeeding experience, contributing to timely initiation [40, 43].

Other factors significantly associated with timely breastfeeding initiation included antenatal visits, marital status, type of birth attendant, and prelacteal feeds. Greater odds of timely breastfeeding initiation were observed among women residing in the middle wealth index; however, limited research exists to explain the correlation of household income and timely breastfeeding initiation. A higher number of antenatal care visits (1–3 and 4 +) were significantly associated with timely breastfeeding initiation among urban women in the stratified analysis. Similar findings were noted in India and Ethiopia, suggesting greater antenatal care visits provide more education and support for early breastfeeding [44, 45]. Married women and those receiving ‘husband support’ may be more likely to timely and exclusively breastfeed due to perceived additional support from their partner [46, 47]. In our study, urban women had greater initiation rates when assisted by a traditional birth attendant, suggesting that their services promote breastfeeding and reduce costs compared to physician or midwife services [48].

Employed women had a lower likelihood of timely breastfeeding initiation, consistent with a systematic literature review conducted in South Asia [49], suggesting women may not feel supported in breastfeeding as they intend to return to work. One of the largest industries in Cambodia, employing mostly young women, is the garment industry [50]. Maternity protection laws in Cambodia grant 90 days for maternity leave, affords one hour per day is paid time off for breastfeeding, and upon returning to work, specifies that duties are lighter for two months [50]. Most women report receiving paid time off to breastfeed, however, most women lack a childcare center at or near the factory [50]. Cambodian law states that factories employing over 100 women must provide a nursing room, however compliance is low, and women report abstaining from bringing their infants to work [50]. Employer incentives could be offered to companies complying with laws protecting maternity rights.

Women offering prelacteal feeds had greater odds of not practicing timely breastfeeding initiation regardless of household wealth index and place of residence. Women residing in rural areas who offered prelacteal feeds had the lowest odds of timely breastfeeding initiation. Although national public health breastfeeding campaigns have helped stabilize breastmilk substitute use with no increase since 2005, limited resources inhibit the continuation of communication campaigns [21]. In the absence of such campaigns, breastmilk substitute companies may increase illegal promotion among vulnerable populations [21], such as those of rural settings without regular media exposure.

Choosing to offer prelacteal feeds, may also be explained by socio-cultural differences between urban and rural areas when considering characteristics and influences of the individual, her household, and community [51]. In May 2014, a press release published in Cambodia called for improved commercial adherence to the WHO and UNICEF International Code of Marketing of Breast-Milk Substitutes and Cambodia’s Sub-Decree 133 [52], whereby the benefits and superiority of breastfeeding must be clearly explained and advocated [53, 54]. This press release cited 113 different breastmilk substitutes being marketed in Phnom Penh with none of them fully compliance with Sub-Decree 133 [52]. In urban settings, such as Phnom Penh, breastmilk substitutes or prelacteal feeds, includes infant/starter formula (indicated for birth to five months of age) [17], while the CDHS includes breastmilk substitutes as formula, sweetened condensed milk, other canned milk usually thinned with water, or watery rice porridge (borbor) [19]. Differences in type of breastmilk substitutes offered is not discernable between rural and urban populations of Cambodia. However, the extensive availability and variety of breastmilk substitutes may contribute to their use in rural and private health centers, as the private health sector continues to market them aggressively [21].

The strength of this study is the large, representative sample of Cambodian women and the availability of various potential confounders for adjustment in the multivariable models. Additionally, all data were collected using a well-establish, standardized and rigorous methodology implemented by the DHS program. Limitations of this study include the cross-sectional design preventing the determination of causation. The role of various skilled birth attendants and health professionals regarding breastfeeding was not captured; therefore, we were unable to determine the extent of breastfeeding counseling and support in the prenatal and early postpartum periods. Additionally, results may have been influenced by self-report and social desirability biases. Lastly, the data lacked information regarding the administration of prelacteal feeds specifically in the first hour, precluding determination of the impact of prelacteal feeds on breastfeeding in the first hour after birth.

## Conclusions

In a representative sample of Cambodian women, household wealth index and residence moderated the association between place of birth and timely breastfeeding initiation. Home births were associated with a lower likelihood of timely breastfeeding initiation, predominantly among women residing in poor households and rural areas. Birth in private facilities was associated with a lower likelihood of timely breastfeeding initiation among urban residents. These findings suggest the need for context-specific interventions, such as incentive schemes for health professionals and health facilities, including employers of young women, along with greater partnership among private and public health sectors are needed to improve timely breastfeeding initiation in Cambodia.

## Supplementary Information

**Additional file 1.** 

## Data Availability

All data generated during this study are included in this published article. Supplementary information files of data, as well as the secondary data analyzed during the current study are available from the corresponding author on reasonable request.
